# Insights into the transition of ductal carcinoma *in situ* to invasive ductal carcinoma: morphology, molecular portraits, and the tumor microenvironment

**DOI:** 10.20892/j.issn.2095-3941.2022.0440

**Published:** 2022-11-01

**Authors:** Weiling Chen, Guimei Wang, Guojun Zhang

**Affiliations:** 1 Department of Breast-Thyroid-Surgery and Cancer Center, Xiang’An Hospital of Xiamen University, School of Medicine, Xiamen University, Xiamen 361101, China; 2 Fujian Key Laboratory of Precision Diagnosis and Treatment in Breast Cancer, Xiang’An Hospital of Xiamen University, Xiamen 361101, China; 3 Key Laboratory for Endocrine-Related Cancer Precision Medicine of Xiamen, Xiang’An Hospital of Xiamen University, Xiamen 361101, China; 4 Xiamen Research Center of Clinical Medicine in Breast & Thyroid Cancers, Xiang’An Hospital of Xiamen University, Xiamen 361101, China; 5 Department of Pathology, Xiang’An Hospital of Xiamen University, Xiamen 361101, China

Breast cancer is posing an increasing burden and has become the cancer with the highest incidence among in women in China. The most common histological subtype of breast cancer is invasive ductal carcinoma (IDC)^1,2^. Ductal carcinoma *in situ* (DCIS) is a pre-cancerous lesion that may give rise to IDC. DCIS is a highly heterogeneous group of lesions consisting of 5 main types, which differ in clinical presentation, histologic features, biomarker profiles, genetic abnormalities, progression potential, and clinical outcomes. When cancer cells invade through the basal membrane, they acquire the ability to metastasize. This process is usually accompanied by many genetic and epigenetic changes in tumor suppressors and oncogenes.

Genetic and epigenetic changes have been identified between DCIS and IDC lesions through molecular profiling and qRT-PCR. The clonal evolution from *in situ* to invasive carcinoma reveals genomic alterations during this transition. Although this topic is controversial, genetic and epigenetic changes have been found to occur in early stages of the transition from normal breast tissue to DCIS^3,4^. Laser capture micro-dissected tissue from pure DCIS and pure IDC has defined key gene expression profiles in the epithelial or stromal compartments associated with disease progression^5^. The most significant alterations in gene expression are observed in the epithelial compartment. In particular, genes associated with the epithelial-to-mesenchymal transition (EMT) and myoepithelial cell-specific genes are enriched in invasive cancer compared with pure DCIS^6,7^.

Recently, a novel method of highly multiplexed ion beam imaging by time-of-flight (MIBI-TOF) has been developed and used to characterize the tumor microenvironment (TME) structure in triple-negative breast cancer by predicting the composition of immune infiltrates and the expression of immune checkpoint inhibitors^8^. A recent article in *Cell* has applied MIBI-TOF and a 37-plex antibody staining panel to reveal the structure and composition of the TME from DCIS and matched IDC samples^9^. The study has delineated 4 tumor microenvironments on the basis of the location and function of the myoepithelium, fibroblasts, and immune cells. The study found more significant myoepithelial disruption in patients with DCIS who did not develop IDC than in those who did, thus suggesting a protective role against recurrence.

In this editorial, we discuss the morphological changes and the evolution of gene expression, including genetic and epigenetic changes in the EMT and microenvironment, during the transition from DCIS to IDC. Furthermore, we emphasize the discovery of changes in the structure and composition of DCIS and IDC stroma recently identified by MIBI-TOF.

## Pathology and morphological changes during the transition from DCIS to IDC

Most DCIS lesions present without symptoms, and very few present with pathological nipple discharge. Mammography often reveals microcalcification, which is relatively more common in DCIS with necrosis, a feature of the more biologically aggressive forms of breast cancer *in situ*. DCIS consists of 5 main types: comedo, solid, cribriform, micro-papillary, and papillary. DCIS with a low nuclear grade often shows cribriform, micro-papillary, or solid architecture, and exceeds 2 mm in size or involves at least 2 ducts. In contrast, DCIS with a high nuclear grade has markedly pleomorphic and misshapen nuclei, with irregular nuclear membranes, coarse chromatin, and prominent nucleoli. Mitoses are often seen and can be atypical, showing clear cytological atypia. Long-term follow-up studies have found that some DCIS cases without treatment may develop into invasive carcinomas, wherein the tumor cells infiltrate the stroma as the myoepithelial cell layer and basement membrane are destroyed^10^.

**Figure 1:**
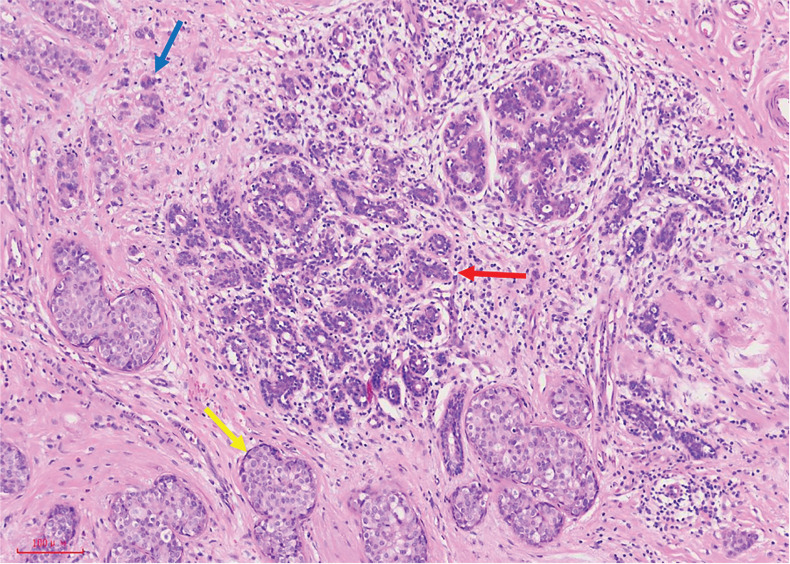
Microscopic pathological morphology of normal terminal duct lobular units, DCIS, and IDC: normal terminal duct lobular units (red arrow) with surrounding DCIS (yellow arrow) and small areas of IDC (blue arrow).

IDC, the most widespread phenotypic subtype of all breast cancers, accounts about 80% of all diagnosed breast cancers^11^. Three categories have been described for DCIS-associated invasive ductal cancers of the breast: microinvasive carcinoma, extensive intraductal component (EIC) of breast cancer, and invasive ductal carcinoma with a predominant intraductal component (IDC with PIDC). Microinvasive carcinoma is defined as an invasive breast carcinoma that is ≤ 1 mm in size and includes 1 or more areas of invasive carcinoma, each no more than 1 mm in the largest dimension. Schnitt et al.^12^ have defined EIC by the presence of 25% or more DCIS in an invasive breast carcinoma. Residual lesions remaining after wide local excision of breast cancer are more likely to be detected when the tumor contains an EIC. IDC with PIDC was first defined in 1981 as IDC with an intraductal component at least 4 times larger than the invasive component **(Figure 1)**.

In general, normal myoepithelium has tumor-suppressive properties and inhibits the progression of DCIS to IDC. In contrast, a growing number of studies have shown that tumor-associated myoepithelial cells have tumor-promoting effects. Recent studies have indicated that normal breast myoepithelial cells are cuboid or spindle shaped, and are present in numbers approximately equal to those of duct epithelial cells. However, the number of myoepithelial cells is diminished in DCIS, and these cells are absent in invasive lesions. Patients with DCIS who do not develop IDC have more severe myoepithelial destruction, myoepithelial hyperplasia (% Ki67), stromal mast cells, and CD4 T cells, thus suggesting that this process may protect against recurrence^9^. The absence of basement membrane material and myoepithelial cells around the nests of tumor cells defines an invasive process.

## Evolution of gene expression during the transition from DCIS to IDC

### Genetic alterations

More than 90% of the gene expression changes observed between normal tissue and IDC occur in early stages of the transition from normal to DCIS. When DCIS and IDC coexist in the same lesion, their genetic aberrations are very similar, demonstrating a high genomic concordance of synchronous DCIS and IDC. DCIS and IDC components from the same patient are often strongly related, on the basis of gene expression and gene copy number aberrations^4^. A meta-analysis of 38 studies has found that DCIS and IDC share a common genetic susceptibility^13^. These studies have demonstrated that pre-invasive lesions and IDC of the same histological grade display remarkably similar gene expression patterns; therefore, identifying gene signatures that robustly distinguish between DCIS and IDC pathological stages may not be possible.

Although DCIS and IDC appear genetically similar, some qualitative differences have been observed between matched DCIS and IDC samples **(Table 1)**. HER2 epidemiology has revealed significant genomic differences between adjacent invasive tumors and *in situ* lesions. Moreover, pure DCIS has higher rates of HER2 overexpression than IDC^14^. Some small studies have reported high-level amplification of *C-MYC* in the invasive component between DCIS and IDC^17^. We previously demonstrated that bcl-2 is expressed in most normal ductal epithelial cells, and its expression gradually decreases during the development of breast cancer, *i.e.*, during the progression from intraductal to invasive carcinoma^15^. p53 expression may occur early in breast cancer development during this progression^15^. Reports have suggested that a single genetic alteration is unlikely to govern the progression of pure DCIS to IDC, whereas shared genetic alterations are likely to predispose some DCIS cases to progress to IDC.

**Table 1:** Genetic alterations from DICS to IDC

Gene symbol	Upregulation	Downregulation	Functions	Reference
*HER2*		√	Unknown	^14^
*BCL-2*		√	Cell proliferation and apoptosis	^15^
*ANAPC13*		√	Cell proliferation and apoptosis	^16^
*CLTCL1*		√	Miscellaneous	^16^
*ADFP*		√	Unknown	^16^
*ARHGAP19*		√	Signal transduction	^16^
*C-MYC*	√		Unknown	^17^
*CXCL9*	√		Signal transduction	^16^
*MED10*	√		Transcriptional regulation	^16^
*MARCH8*	√		Miscellaneous	^16^
*NOX4*	√		Cell proliferation and apoptosis	^16^
*P4HA1*	√		Metabolism	^16^
*HN1*	√		Unknown	^16^

### Epigenetic modifications

Several recent studies have focused on defining epigenetic changes, such as DNA methylation, histone modifications, and miRNA expression, that ultimately affect gene expression during the transition from DCIS to IDC. The studies have concluded that most of the aberrations in epigenetic profiles are observed early in the pre-invasive DCIS stage; however, the variations between DCIS and IDC are relatively minor^3^.

Previous studies have suggested that DNA methylation increases during the transition from normal breast tissue to DCIS, but occurs at a similar frequency in DCIS and IDC^18^. The alterations in DNA methylation during the transition from normal epithelium to DCIS may play important roles in early breast carcinogenesis.

Histone modifications have also been considered during breast cancer progression. Elsheikh et al.^19^ have detected a series of histone lysine acetylations in well-characterized human breast carcinomas (*n* = 880) by using tissue microarrays. Acetylation of H4K16ac was absent in most samples, thus suggesting that loss of H4K16ac acetylation may be an early event in breast cancer^19^. Transcript and protein levels of the histone lysine N-methyltransferase EZH2 are elevated in DCIS lesions but largely absent from normal tissue^20^. Similarly, studies have shown that histone deacetylase expression and activity may be altered between normal and DCIS model cell lines, whereas no change is observed between DCIS and IDC; thus, these changes may be early events in progression^21^. In agreement with observations of DNA methylation, these studies have indicated that histone modification may be an early event in breast cancer progression.

The effects of miRNAs on mRNA expression and function on the pathogenesis of breast carcinogenesis have been clarified in the past decade. miRNA analysis has indicated that normal breast samples can be distinguished from most DCIS and IDC by increases in miR-21 and decreases in multiple miRNA families; most miRNA alterations arise early in DCIS^22^. miRNA profiles established for the transition from normal breast epithelia to DCIS remain essentially unchanged during the progression from DCIS to IDC. These findings have revealed the importance of identifying candidate miRNAs in predicting the risk of DCIS progression.

## Changes in the cellular signaling pathways and microenvironment during the DCIS to IDC transition

### Cellular signaling pathways

Studies are increasingly indicating that a series of cell signaling pathways may be involved in the transition from DCIS to IDC. Findings from Lo and colleagues support the paradigm that altered DCIS-associated myoepithelial cells promote the invasive progression of DCIS into IDC *via* TGFβ signaling activation^23^. A study has revealed that the MAPK-interacting serine/threonine-protein kinase 1 (MNK1)/NODAL signaling axis is a key molecular pathway regulating the progression of DCIS to IDC^24^. In particular, significant alterations in EMT related pathways and gene expression have been found in the progression of DCIS^6^. Choi et al.^7^ have shown that altered expression of mesenchymal markers (*e.g.*, loss of E-cadherin and altered β-catenin) is greater in invasive carcinomas than in pure DCIS. Together, these data demonstrate that cellular signaling pathways, such as the TGFβ pathway, may play important roles in the progression of DCIS to IDC.

### Microenvironment

The unexpected lack of genomic evolution between DCIS and IDC has led researchers to focus on the potential role of the stromal microenvironment in mediating the transition from DCIS to IDC. Cancer-associated fibroblasts (CAFs) have been found to play a crucial role in the progression of DCIS to IDC. During this transition, mast cells, resting fibroblasts, and normal fibroblasts in the stroma are diminished, whereas normal fibroblasts in the primary DCIS samples are replaced by CAFs in subsequent invasive breast cancer in the same patients^9^. In addition, studies have shown that during the transition from DCIS to early IDC, host lytic T cells interact with and destroy tumor tissue, thus leading to an adaptive upregulation of PD-L1 for tumor protection against immune destruction^25^.

Several studies have examined the pre-invasive influence of the extracellular matrix (ECM) and non-tumoral cells on DCIS, and revealed that substantial changes occur in the TME during progression from DCIS to IDC. Vargas and co-workers^5^ have observed that ECM remodeling factors are differentially expressed between DCIS and IDC. In another study, Gil Del Alcazar et al.^26^ have analyzed the composition of leukocytes in normal breast tissue, DCIS, and IDC, and found a significant decrease in CD8+ signatures and fewer activated GZMB (granzyme B)+CD8+ T cells in IDC than in DCIS. The T-cell receptor clonotype diversity was also significantly higher in DCIS than IDC, thus suggesting differences in tissue- and tumor-specific T cells and neutrophils. With improvements in capturing and detecting molecular differences in single cells, we expect new progress to advance understanding of the role of the TME in promoting the progression of DCIS to invasive disease.

## Changes in the structure and composition of DCIS and IDC stroma identified by MIBI-TOF

**Figure 2:**
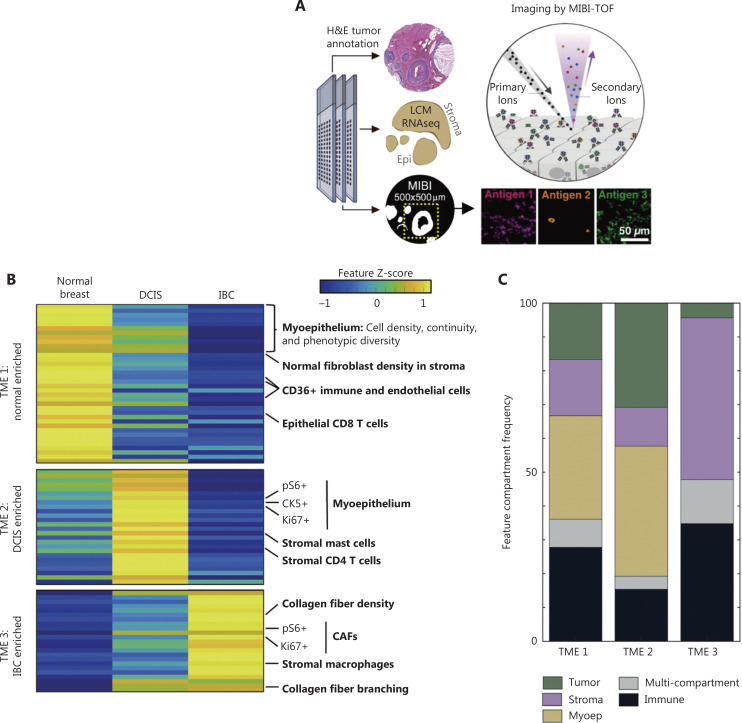
Transitions to DCIS and IDC are marked by coordinated changes in the TME^9^. (A) Depiction of the parallel tissue analysis methods used in this study, including hematoxylin and eosin (H&E) histochemical staining, laser-capture microdissection (LCM) of stroma and epithelium with RNA-seq, and MIBI-TOF, including an overview of the MIBI-TOF workflow. (B) Heatmap of the distinguishing features prevalent in normal breast tissue, DCIS, and recurrent invasive breast cancer (IBC) samples. K-means clustering separated the features into 4 groups with distinct feature-enrichment patterns in the tissue states, including those highest in normal tissue and low in IBC (TME1: normal enriched); those highest in DCIS (TME2: DCIS enriched); and those highest in IBC and low in normal tissue (TME3: IBC enriched). The features are organized by descending false-discovery rate Q values within each TME. The colors indicate the mean over tissue states, Z scored per feature across tissue states. (C) Area plot of the distribution of the cellular compartments of the distinguishing features in each TME cluster.

To understand how DCIS structure and single-cell function are interrelated, Keren et al.^27^ developed a multiplex imaging platform, MIBI-TOF, to analyze a large cohort of archived human tissue samples covering the spectrum of breast cancer progression, from *in situ* to invasive disease, in a spatially resolved manner. MIBI-TOF has also been used to identify phenotypes governing the TME structure in triple-negative breast cancer (TNBC)^8^. More recently, the same group has used MIBI-TOF and a 37-plex antibody staining panel to comprehensively define the cellular composition and structural characteristics of normal breast tissue, DCIS, and IDC^9^. This later work has indicated that the epithelial and stromal tissue compartments of DCIS are composed predominantly of epithelial cells and fibroblasts, respectively, each comprising 4 major phenotypic subsets. All phenotypic subsets of IDC and normal breast tissue are present in DCIS, such that DCIS cannot be differentiated from IDC solely on the basis of the presence of discrete cell types **(Figure 2)**. However, coordinated changes in the TME have been found to mark the transitions to DCIS and IDC. The distinguishing features indicate 4 interpretable TME signatures for specific breast tissue states: TME1, TME2, and TME3, distinguishing normal, DCIS, and IDC samples, respectively; and TME4, consisting of features specifically absent from the DCIS samples.

**Figure 3:**
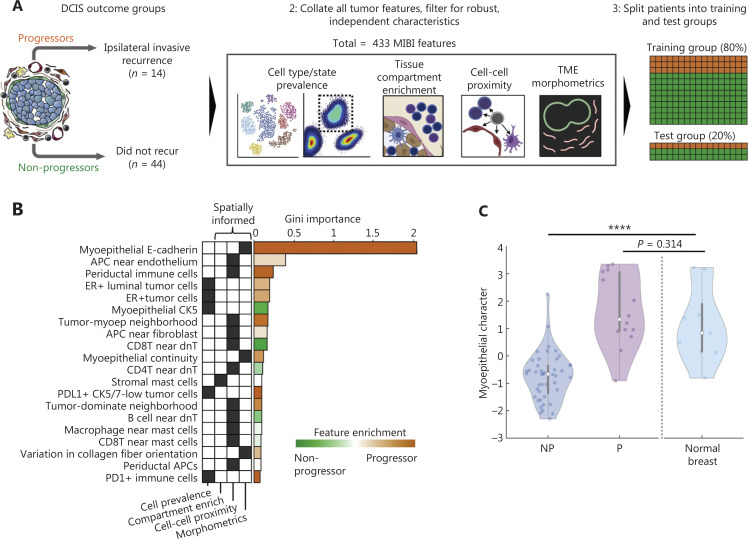
Identifying DCIS features correlated with the risk of invasive progression and phenotypic changes between progressors and non-progressors^9^. (A) Schematic of the outcome groups for primary DCIS: progressors (P), recurrence with ipsilateral IDC; non-progressors (NP), no recurrence within 11 years of follow-up. MIBI features (*n* = 433) of numerous feature classes were used to train a random forest classifier to differentiate P and NP samples. Classifier specificity was tested on a withheld set of 20% of the patients in the test group. (B) Bar plot for the features with top classifier importance, ranked by average Gini importance across 10 unpermuted runs. Orange, enriched in P; green, enriched in NP. The parent feature class for each feature and whether that class leveraged spatial information are shown. (C) Violin plot of the distribution of the linear discriminant analysis-derived “myoepithelial character” values in NP and P tumors and normal breast tissue, assessed with the Kruskal-Wallis test.

This group has also built a random forest classifier for predicting which DCIS cases later progress to IDC. They have also detected myoepithelial breakdown and phenotypic changes between progressors and non-progressors. Typically, the non-progressors are enriched in parameter ontologies associated with hypoxia, glycolysis, stromal immune density, and desmoplasia/remodeling of the ECM. In contrast, the high myoepithelial character scores typically seen in progressors are enriched in immunoregulatory marker expression (*PDL1*, *IDO1*, *COX2*, and *PD1*) in tumor and immune cells **(Figure 3)**. This method may serve as an important tool for studying the DCIS to IDC transition and developing drugs targeting key molecules during this process.

The researchers have also attempted to mitigate cell classification and clustering by using a hybrid approach using FlowSOM to hierarchically stratify cell populations on the basis of well-vetted multiparameter phenotypes. Correlations have been found between low myoepithelial integrity and increased stromal immune infiltration, collagen deposition, and CAFs; however, whether myoepithelial breakdown triggers this correlation in the TME or *vice versa* has not been determined. This work has several limitations, such as the small patient cohort, imaging methods, data analysis, and interpretation of the results.

## Perspective

In the transition of carcinoma from *in situ* to invasive, the specific mechanisms or manifestations involved may differ among types of cancers. Herein, we focused on the recent progress in understanding the progression of DCIS to IDC. In particular, we discussed the genetic and epigenetic changes occurring during the transition from DCIS to IDC. Many genetic profiles and epigenetic modifications appear to occur in early stages of DCIS rather than in the transition from DCIS to IDC, even in the atypical hyperplasia stage.

MIBI-TOF has identified cell clusters in tissue sections of epithelial, CAF, and immune cells, and has enabled relatively accurate cell enumeration in 3-dimensional images in the TME of both DCIS and IDC. Phenotypic changes and myoepithelial breakdown have been determined with 4 different TME signatures, thus distinguishing DCIS that progresses from DCIS that does not progress to IDC. Future studies are needed to confirm this relationship in a larger independent cohort of patients with DCIS that progressed to invasive disease, and to investigate its association with stromal desmoplasia in functional models.

In summary, these novel findings provide new insights into the progression of DCIS to IDC, and might enable the development of tailored clinical management, particularly immunomodulatory treatment, for people with DCIS in the future.
